# Anatomical and Visual Outcomes after LASIK Performed in Myopic Eyes with the WaveLight® Refractive Suite (Alcon® Laboratories Inc., USA)

**DOI:** 10.1155/2020/7296412

**Published:** 2020-10-03

**Authors:** Imene Salah-Mabed, Sarah Moran, Emmanuelle Perez, Guillaume Debellemanière, Damien Gatinel

**Affiliations:** ^1^Department of Anterior Segment and Refractive Surgery, Rothschild Foundation, Paris, France; ^2^Center of Expertise and Research in Optics for Clinicians, Stanford, CA, USA

## Abstract

**Purpose:**

To evaluate changes in corneal anatomy and quality of vision following LASIK refractive surgery for mild to high myopia using the WaveLight® Refractive Suite (Alcon® Laboratories Inc., USA). *Setting*. Rothschild Foundation, Paris, France.

**Design:**

Prospective interventional case series.

**Methods:**

We examined 60 myopic eyes (average SE −4.5 D, from −9.3 to −0.75 D) of 30 patients from 21.3 to 38.7 years old. Pachymetry, keratometry, *Q* factor, corneal aberrations, visual acuity (VA), contrast sensitivity, dry eye assessment, and quality of vision were measured preoperatively, one day (D1), and 1, 3, and 6 months postoperatively.

**Results:**

6 months postoperatively, keratometry became flatter, and the *Q* factor became more oblate (from −0.18 ± 0.08 to +0.19 ± 0.06). Pachymetry decreased by 117.9 ± 62.2 *µ*m at D1 and increased by 37.87 ± 32.6 *µ*m between D1 and M6. Refraction was emmetropic at D1 and remained stable thereafter. Six months after surgery, VA was slightly but nonsignificantly improved (<0.05 log MAR), whereas contrast sensitivity remained unchanged. Quality of vision was not affected by surgery and was more related to dry eye symptoms than to corneal HOAs (*r*^2^ = 0.49; *p* < 0.001 vs. *r*^2^ = 0.03; *p* < 0.001).

**Conclusions:**

LASIK surgery for moderate to high myopia, performed with the WaveLight® Refractive Suite, showed good postoperative outcomes, with demonstrated safety, predictability, efficiency, and stability. This is probably due to well-controlled spherical aberration and the use of large optical zones. Besides, we can assume that the patients' quality of vision depends more on the postoperative dry eye disease generated by the laser than on the induced HOAs.

## 1. Introduction

LASIK [1, 2] is an increasingly popular surgical option for the correction of myopia as demonstrated by the rising numbers of these procedures being performed worldwide. The technique involves use of a femtosecond laser to create a hinged flap, which is then folded back to allow photoablation of the exposed stroma using an excimer laser. In myopic LASIK, stromal tissue is removed, resulting in flattening of the central corneal curvature, which in turn decreases the excessive refractive power of the eye.

In recent years, an increasing amount of research has been focused on the assessment of quality of vision after LASIK refractive surgery [3–7].

The aim of refractive surgery is to improve visual outcomes and to reduce dependence on spectacles or contact lenses. In the 1990s, many studies were published on the correction of myopia with LASIK [8, 9] reporting low predictability, significant regression, and induced night vision disturbances [10, 11]. These issues were due in large part to the use of small optical zones [12, 13] and nonaspheric Munnerlyn ablation profiles leading to significant induced spherical aberrations [14]. In the 2000s, other studies reported that LASIK was a safe and predictable method to correct moderate to high myopia [15, 16]. Indeed, these studies show a high success rate, reflected by favourable functional outcomes [17–20], and high physician and patient satisfaction [21].

Several studies report satisfaction rates of *c*. 90% after LASIK [15, 22, 23]; however, others report dissatisfaction, suggesting room for future improvement [24].

Most published studies evaluate the visual outcomes of LASIK in terms of visual performance (visual acuity, contrast sensitivity, and depth of focus) [25, 26]. Other studies describe the microstructural changes induced in the stroma and Bowman's layer in vivo using confocal microscopy [27]. However, many questions remain regarding the biological response of the cornea to the ablation process [28]. These microstructural disturbances of the corneal stroma may result in wavefront aberrations [29]. Recently, with the development of newer techniques, we can measure the optical wavefront after refractive surgery. Studies have revealed that although refractive errors are reduced, higher-order aberrations are generally induced [30, 31]. Along with other technical advances (eye trackers, small-spot lasers, etc.), the accurate measurement of ocular wave aberrations has opened doors for potential improvements of LASIK, in particular through customized treatments for each patient eliminating low- and high-order aberrations in the eye [32, 33].

It has been shown that analysis of the total wavefront error of the eye reflects the most complete measurement of retinal image quality, which is directly related to the visual performance [34, 35]. Although the impact on the visual performance is not fully understood, wavefront-error data have been extensively used as an objective parameter for the quality of vision in theoretical models and in clinical trials [17, 18, 36, 37]. That said, it is desirable to establish robust and clinically meaningful correlations between the results of wavefront analysis and subjective quality of vision.

This study describes the anatomical and visual outcomes of myopic LASIK performed with the WaveLight® Refractive Suite (Alcon® Laboratories Inc., USA) which includes a FS200 femtosecond laser and an EX500 excimer laser. We present anatomical changes, biomechanical corneal response (both anterior and posterior surfaces), visual performance (visual acuity, contrast sensitivity, and depth of focus), total and corneal aberrations, and patient satisfaction before and after LASIK. We also aimed to correlate all these parameters to obtain a more complete view of the present outcomes of LASIK surgery in moderate to high myopia with the aforementioned devices.

## 2. Patients and Methods

### 2.1. Patients

This study included 60 eyes of 30 patients undergoing LASIK surgery for myopia at the Rothschild Foundation from May 2015 until June 2016. All patients underwent complete ocular assessment prior to surgery, including cycloplegic refraction, slit lamp, and fundus examination. Preoperative corneal topography was performed with OPD-Scan® III (Nidek®, Japan) and the Orbscan IIz® (Bausch & Lomb®, USA).

Patients presenting with corneal disease or other ocular pathologies (amblyopia, glaucoma, cataract, retinopathy, and strabismus), those with evidence of subclinical keratoconus, or those with a history of ocular surgery were excluded from the study. We also excluded patients whose eyes tested positive for keratoconus (KC) or keratoconus suspect (KCS) diagnosed by the corneal navigator neural network, which uses Klyce and Maeda indices on OPD-Scan® III (Nidek®, Japan). Patients who had worn rigid gas-permeable lenses in the 12 months prior to examination and those who had worn soft contact lenses in the 3 weeks prior to surgery were also excluded.

We included myopic patients older than 18 years with otherwise unremarkable ophthalmic histories. The choice of the LASIK technique was justified by the presence of a thick cornea (defined as a residual stromal bed higher than 300 *µ*m after subtracting the sum of the planned LASIK flap and laser ablation thickness) and the presence of a regular corneal surface diagnosed with an objective method based on Placido disk-derived data for the detection of eyes at the risk of ectasia [38].

All patients provided written informed consent. The study and data acquisition were achieved with the approval from the Rothschild Foundation's institutional review board. Informed consent was obtained from each patient after he/she voiced understanding about the purpose and the procedures in the study in accordance with the Declaration of Helsinki.

### 2.2. Surgical Procedure and Treatment Planning

The 60 eyes enrolled in this prospective study underwent uncomplicated primary LASIK performed by the same experienced surgeon (DG) using the same refractive surgery platform (FS200 femtosecond laser and EX500 excimer laser). WaveLight FS200 femtosecond laser system is a low-energy and high pulse frequency laser that emits laser pulses with the duration of 350 fs at a wavelength of 1,050 nm and a pulse repetition rate of 200 kHz.

Flap creation was performed with the FS200 femtosecond laser, using standard treatment settings (9.2 mm flap diameter and 110 *µ*m flap thickness).

A Munnerlyn algorithm-based photoablation [39] was performed with the EX500 excimer. A standard aspheric ablation profile was planned with a plano target (at the corneal plane) refraction. The average optical zone was 6.5 mm with a transition zone of 1.25 mm. For some subjects, because of a greater deviation between the pupillary axis and the visual axis (kappa angle) [40], preoperative corneal vertex and pupillary axis were measured by WaveLight® Topolyzer^TM^ VARIO (Alcon® Laboratories Inc., USA) linked with the EX500 excimer laser. A valid assumption is to consider that the optimal centration for corneal refractive surgical procedures may be located close to or midway between the corneal vertex (first Purkinje image) and the pupil centre [41, 42]. However, in some eyes, the distance between these points can be as high as 400 *µ*m. This reflects the presence of a large kappa angle. Defining the proper axis for centration may be of critical importance in eyes with a large distance between the pupil centre and the corneal vertex. EX500 excimer laser software enables centration of the excimer profile of ablation from the pupil centre (0%) to the corneal reflex (100%), or in between, by a 10% step distance along the line joining the pupil centre to the corneal reflex. For this reason, all treatments were centred equidistant between the pupil centre and the corneal vertex (50%) in each patient.

### 2.3. Preoperative and Postoperative Evaluation

Ophthalmologic examination performed on all patients preoperatively included manifest refraction, cycloplegic refraction, noncontact intraocular pressure evaluation, slit-lamp microscopic evaluation of the anterior segment, and dilated fundoscopy. Preoperative examination included evaluation of pachymetry, keratometry, elevation and curvature topography analysis with Orbscan IIz® (Bausch & Lomb®, USA), wavefront aberrometry (root mean square on the 5.5 mm pupil), and corneal asphericity (at 6 mm diameter) analysis with the OPD-Scan® III (Nidek®, Japan) topographer (Nidek, Inc., Fremont, CA). Corneal asphericity and corneal and total ocular aberrations were analysed according to the Optical Society of America (OSA) recommendations [43]. Dry eye assessments were evaluated using the corneal tear film break up time (BUT) index.

**Figure 1:**
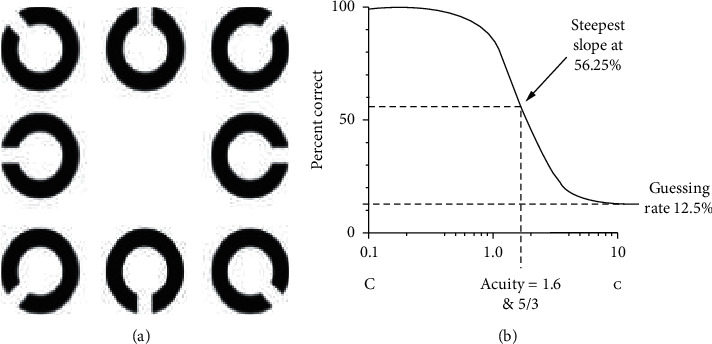
(a) Landolt rings displayed during the visual acuity test. 8 orientations were possible: left, top left, top, top right, right, bottom right, bottom, and bottom left. (b) Psychometric function used by the Freiburg test. The probability of correct answers depends on the size of the optotype. Visual acuity is 16/10 (−0.2 logMAR) [44].

Ten percent and 90% contrast uncorrected distance visual acuity (UDVA) and best corrected distance visual acuity (CDVA) were assessed using FrACT (Freiburg Visual Acuity and Contrast Test) 139 software at 4 meters monocularly and binocularly. This corresponds to the presentation of Landolt rings at 8 positions (Figures 1(a) and 1(b)).

Because the patients would experience a minimizing effect from myopic correction of the trial lenses, magnification adjustment was made to the corneal plane so as to properly compare preoperative and postoperative vision. Visual acuities were adjusted according to the patient's refractive correction. The trial lens vertex distance of 17 mm was used to calculate relative magnification (RM):

(1) $$\begin{array}{c} \text{RM}=1-\text{hFs,} \end{array}$$

where *h* is the difference between the corneal and spectacle plane (vertex distance in meters) and Fs the back-vertex power of the corrective lens at the spectacle plane. The following equation was used to convert visual acuity from the spectacle plane to the corneal plane: logMAR_cornea_ = logMAR_spec_–logRM [26].

Photopic corrected distance and uncorrected contrast sensitivity (CCS and UCS) measurements were performed at 12 cycles per degree (cpd), using randomly oriented sinusoidal arrays at 4 meters. Photopic best-corrected and uncorrected contrast sensitivities were also measured with the introduction of glare. To generate glare, oncoming headlights were simulated by attaching 2.5 watt halogen floodlights to each side of the computer screen [26].

A “tolerance to blur” measurement was also performed (corrected sensitivity to blur, CSB). The subjective depth of field criteria used was unacceptable blur. This is the level of blur that the patient would refuse to accept if he had to endure it permanently. The average generally observed is about 1.4 D [45–47]. During the evaluation, the subjects wore their spherocylindrical correction and held in front of their eyes an artificial pupil of 3 mm that was subjectively adjusted to maximize the contrasts.

**Figure 2:**
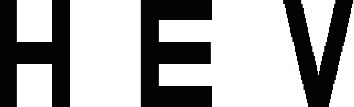
Black-and-white (HEV) images presented for tolerance/sensitivity to blur.

Starting systematically from the clear image (Figure 2) as a reference, defocalization was added to each new slide (0.05 *μ*m or about 0.055D). The subject had to say “stop” as soon as the image was no longer acceptable according to the criterion of unacceptable blur described by Atchison [46, 48].

The illumination of the room where the tests were carried out was about 350 lux. The luminance of the screen that projects the contrast sensitivity, visual acuity, and simulated images for the depth of field measurement has been systematically calibrated to about 100 candelas per *m* [2].

All patients completed two French versions of vision quality questionnaires: (QOV) [49] (range: 0 excellent quality of vision to 100 very poor quality of vision) and Ocular Surface Disease Index (OSDI) which was developed to quantify the specific impact of dry eye disease on vision-targeted health-related quality of life (range: 0 normal to 100 severe dry eye) [50] preoperatively and one, three, and six months postoperatively. The overall OSDI score defined the ocular surface as normal (0–12 points) or as having mild (13–22 points), moderate (23–32 points), or severe (33–100 points) disease.

All these parameters were measured preoperatively on day 1 and 1 month, 3 months, and 6 months postoperatively. Each measured parameter was verified by the examiner prior to recording. All measurements were performed by the same operator (IS).

### 2.4. Statistical Analysis

Statistical analysis was performed with commercial software (SPSS v. 13.0; SPSS Inc., Chicago, IL). We used paired and unpaired Student's *t*-test to compare the outcomes in this population. ANOVA test was also used to compare means. Pearson correlation analyses were also used. A calculated *p* value <0.05 was considered statistically significant. Data are presented as the mean ± standard deviation.

Astigmatism plots were generated using AstigPLOT® software (EB Eye). The average magnitude and the axis of cylinders were computed using vector calculations. The astigmatism plots were represented with a positive cylinder magnitude convention.

## 3. Results

### 3.1. Demographics

60 myopic eyes of 30 patients were included in the study. The mean preoperative spherical equivalent was −4.5 ± 2.2 D (ranging from −9.3 D to −0.8 D). 43 eyes of 60 (72%) had a SE higher (more myope) than −3.00 D, and 3 eyes among 60 had a SE lower or equal to 1 diopter. The mean age was 30.4 ± 4.2 years (ranging from 21.3 to 38.7 years). Data are further detailed in Table 1.

### 3.2. Anatomical Changes

**Figure 3:**
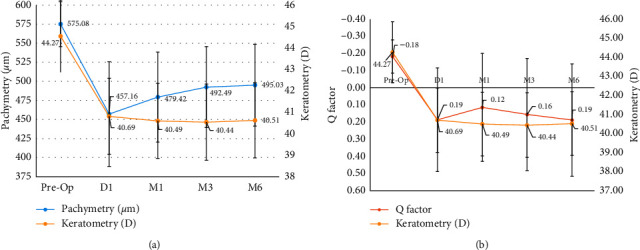
Evolution of anatomical parameters after myopic LASIK. (a) Evolution of pachymetry with regard to keratometry. (b) Evolution of keratometry with regard to asphericity.

Six months postoperatively, the cornea became flatter (44.27 ± 1.61 D preoperatively to 40.51 ± 1.67 D at 6 months postoperatively). There was a significant difference between the average corneal power before and after LASIK (paired *t*-test, *t* = 17.50; *p* < 0.001) (Figure 3(a)).

There was no correlation between the average keratometric power and the patient's age, refractive spherical equivalent, and the initial central mean pachymetry, before and after the surgery (ex: correlation between preop keratometry and age, *r*^2^ = 0.184; *p*=0.160).

The mean corneal pachymetry was 575.08 ± 29.41 *µ*m, 457.16 ± 68.59 *µ*m, 479.42 ± 58.97 *µ*m, 492.49 ± 53.18 *µ*m, and 495.03 ± 53.79 *µ*m, respectively, preoperatively one day and 1 month, 3 months, and 6 months postoperatively. 6 months postoperatively, the pachymetry was significantly lower than preoperatively (paired *t*-test, *t* = 15.03; *p* < 0.001).

**Figure 4:**
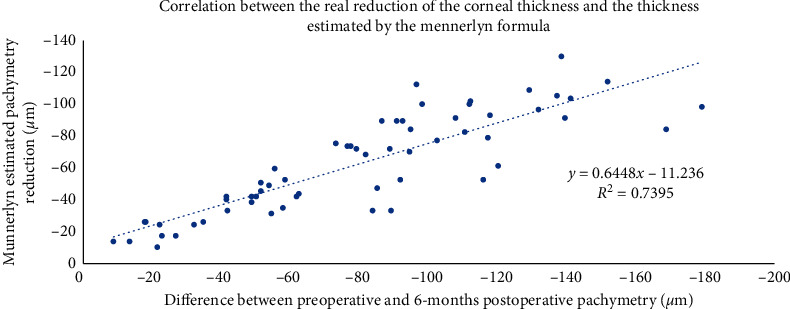
Positive correlation between the real reduction of the corneal thickness and the thickness estimated by the Munnerlyn formula.

Six months postoperatively, the mean decrease in keratometry was 3.76 ± 1.66 D while the mean decrease in pachymetry was 80.04 ± 41.26 *µ*m. The difference in pachymetry at 6 months postoperatively correlated positively (*r*^2^ = 0.74; *p* < 0.001) with the Munnerlyn formula pachymetry estimation (Figure 4).

On day one after LASIK, corneal asphericity as expressed by the *Q* factor became significantly more oblate (*Q* = −0.18 ± 0.10 (SD) (range: −0.38 to 0.05)) preoperatively and *Q* = 0.19 ± 0.30 (SD) (range: −0.29 to 0.98) one day after surgery (*t* = −9.52; *p* < 0.001). There was no significant difference in the *Q* factor at different time points after surgery (*t* = −0.31; *p*=0.98) (Figure 3(b)).

There was no correlation between the preoperative spherical equivalent and the preoperative corneal asphericity measured (*r* = −0.003; *p*=0.98). There was no correlation between the initial mean central pachymetry and the corneal asphericity (*r* = 0.206; *p*=0.18).

### 3.3. Safety and Predictability

#### 3.3.1. Quality of Vision Outcomes

**Figure 5:**
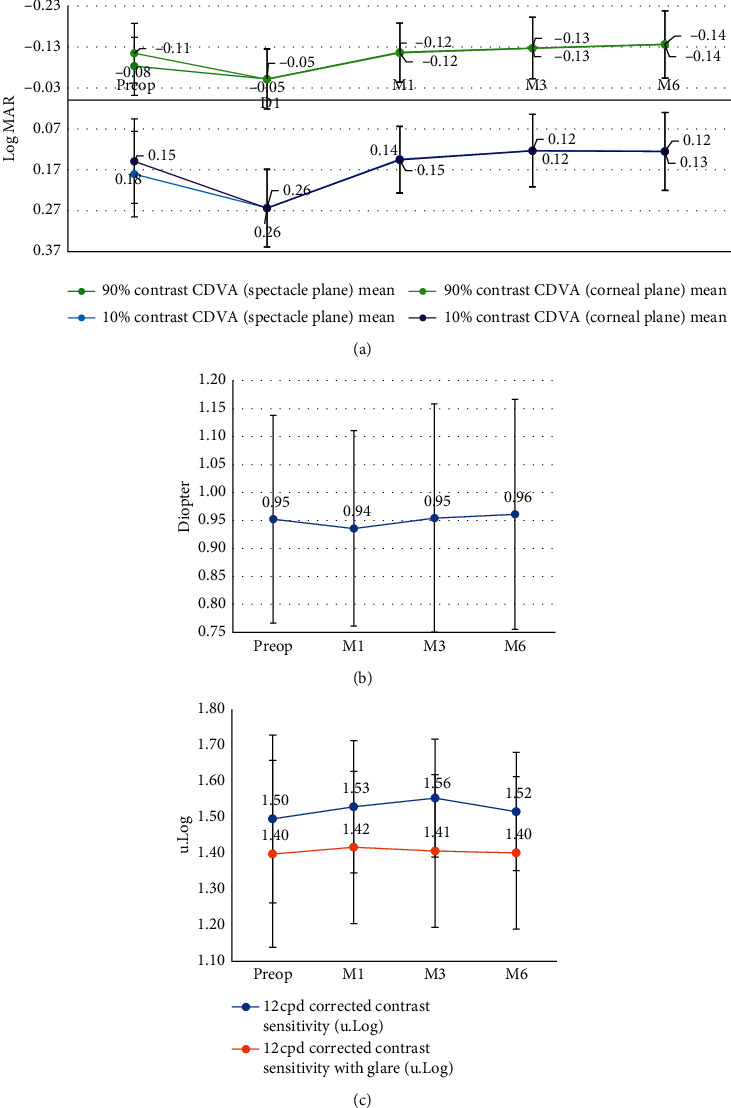
Evolution of visual outcomes after myopic LASIK. (a) Evolution of 90% and 10% contrast CDVA in the spectacle and the corneal planes. (b) Evolution of the corrected sensitivity to blur. (c) Evolution of 12 cpd corrected contrast sensitivity and 12 cpd corrected contrast sensitivity and 12 cpd corrected contrast sensitivity with glare.

Figure 5 shows that, after LASIK, monocular 90% and 10% CDVA increased slightly but not significantly (paired *t*-test, *t* = 2.07; *p*=0.053 and *t* = 1.62; *p*=0.11, respectively), while monocular corrected contrast sensitivity and corrected sensitivity to blur remained unchanged (paired *t*-test, *t* = −0.75; *p*=0.46 and *t* = −0.36; *p*=0.72, respectively). The CCS with glare was lower than the CCS by 0.1 u.log.

Table 2 shows the safety and predictability of LASIK in terms of quality of vision outcomes. Although there was no difference in the quality of vision outcomes (CDVA and CSB) preoperatively between high (cylinder ≥ 1.5 D) and low astigmatic eyes (cylinder < 1.50 D) except for the CCS (where the high astigmatic eyes CCS was smaller than the low astigmatic one (ANOVA, *p*=0.016)), there were differences postoperatively. No significant difference was found between groups in CSB.

#### 3.3.2. Refractive Spherical Equivalent Outcomes and Magnitude of Astigmatism

**Figure 6:**
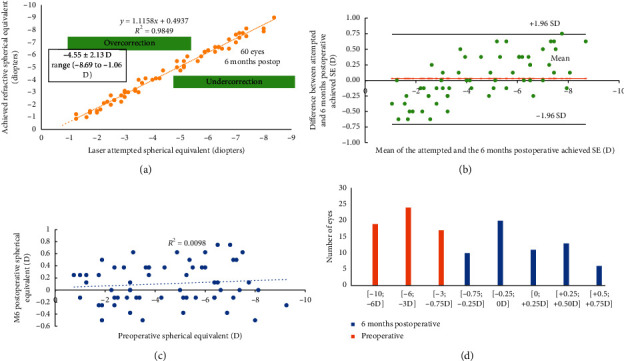
Refractive spherical equivalent (SE) outcomes: distribution of achieved SE outcomes after LASIK at 6 months (a), Bland–Altman distribution of attempted SE (b), correlation between preoperative SE and 6 months postoperative SE (c), and distribution of manifest SE preoperatively and 6 months postoperatively (d).

Figures 6(a) and 6(b) show predictability of the manifest SE (scattergram of attempted versus achieved manifest SE). There was a strong and statistically significant correlation between the laser attempted SE and the achieved SE (*r*^2^ = 0.98; *p* < 0.001). The postoperative SE was independent from the preoperative SE (*r*^2^ = 0.0098; *p* < 0.001) (Figure 6(c)), and there were no statistically significant differences in the SE 6 months postoperatively between high astigmatic eyes and low astigmatic ones (ANOVA, *p*=0.98).  Figure 6(d) displays the distribution of preoperative and 6 months postoperative SE.

Astigmatism is an optical aberration which is mainly caused by the toricity of a refractive surface. Although topography instruments measure toricity (not astigmatism), we will use the terms “astigmatism” and “toricity” interchangeably. The magnitude of the astigmatism was calculated as follows.

**Figure 7:**
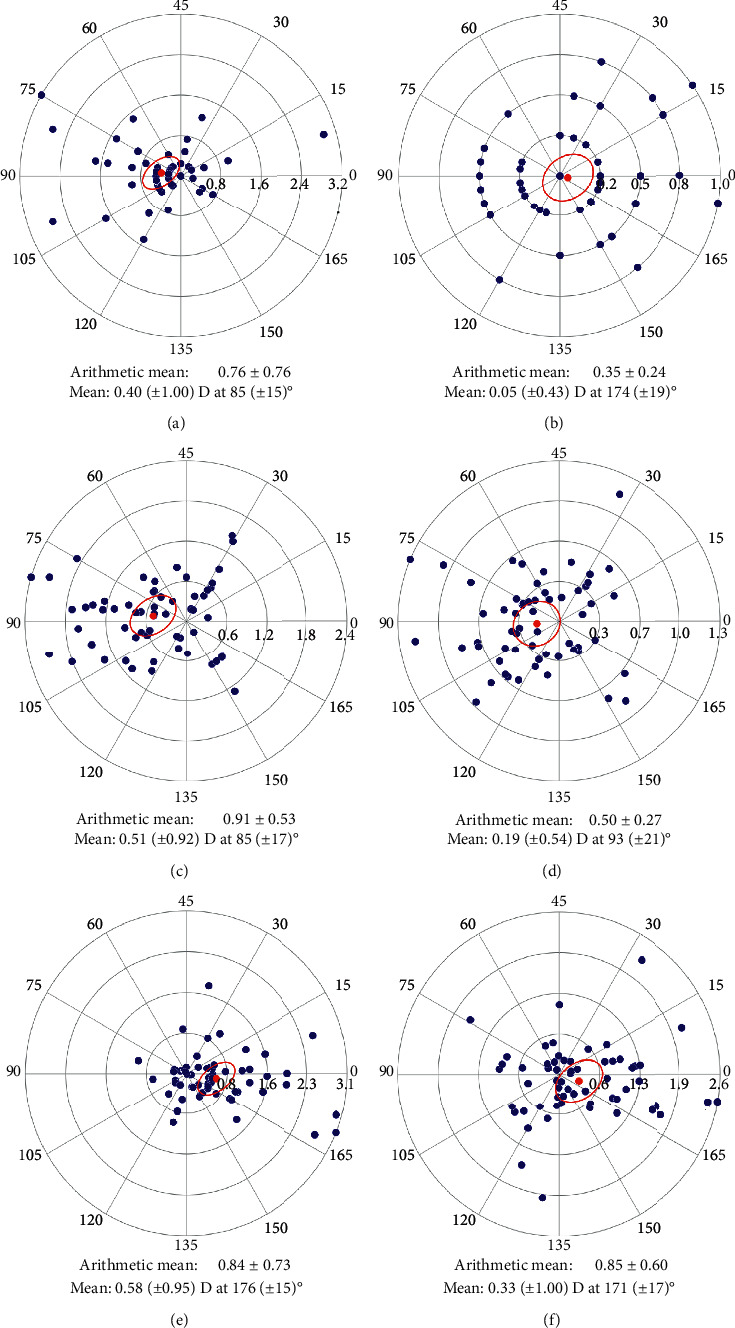
Preoperative refractive astigmatism (a), 6 months postoperative refractive astigmatism (b), preoperative corneal astigmatism (c), and 6 months postoperative corneal astigmatism (d). Difference between preoperative and 6 months postoperative refractive astigmatism (e) and corneal astigmatism (f).

In the 5 mm ring zone, the difference in simulated keratometry (sim-K) of the steepest and the flattest hemimeridians was calculated as the “sim-K difference” by topography software. The magnitude of the astigmatism was computed as the variation between the “sim-K difference” values. The average refractive astigmatism value decreased from 0.40 D preoperatively to 0.05 D 6 months postoperatively. And the corneal astigmatism decreased from 0.51 D to 0.19 D after LASIK. Before and after LASIK, the astigmatism was predominantly with the rule (WTR) except for the total refractive astigmatism which was oriented against the rule at 6 months (Figure 7(b)).

Figure 7 represents the magnitude and orientation of the refractive and anterior corneal astigmatism preoperatively and 6 months postoperatively. We found a difference of 0.58 D (for the refractive astigmatism) and 0.33 D (for the corneal astigmatism) between the two analysed periods.

There was no correlation between the 6-month postoperative cylinder value and the preoperative cylinder (*r*^2^ = 0.0013; *p* < 0.001).

#### 3.3.3. Corneal and Total Aberrations' Analysis on a 5.5 mm Pupil

**Figure 8:**
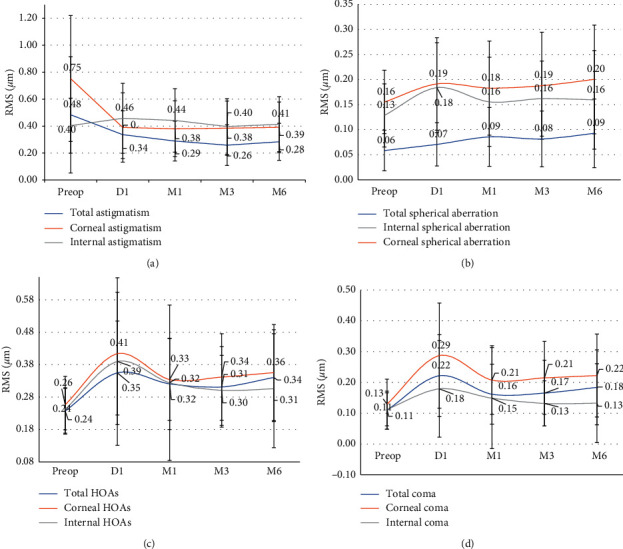
Evolution of RMS ocular total, corneal, and internal aberrations (*µ*m): astigmatism evolution (a), spherical aberration evolution (Zernike SA4 + SA12) (b), high-order aberrations (HOAs: 3rd order and higher) (c), and coma evolution (d).

**Figure 9:**
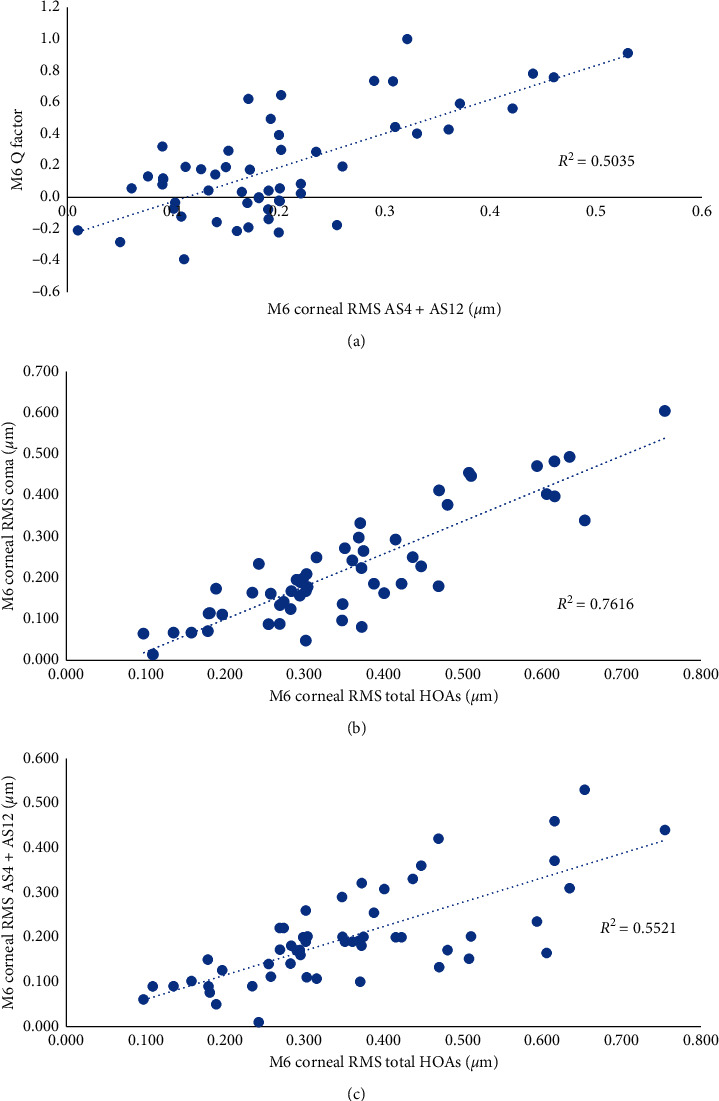
Relationship between spherical corneal aberrations and *Q* factor (a), between corneal HOAs and corneal coma (b), and between corneal HOAs and corneal spherical aberrations (c).

Figure 8 and Table 3 show the very slight but significant increase in total, corneal, and internal ocular aberrations after LASIK surgery. The most important increase in corneal and total HOAs seems to be attributed to the increase of corneal coma (Figure 9). The total spherical aberration increased very slightly but significantly (0.034 ± 0.063; *p* < 0.001).

We found no correlations between total, corneal, and internal spherical aberrations after LASIK and preoperative SE (*r*^2^ = 0.03; *p* < 0.001, *r*^2^ = 0.012; *p* < 0.001, and *r*^2^ = 0.009; *p* < 0.001, respectively). No predictive factor for the increase in postoperative HOAs was found (low *r*^2^, *p* > 0.05). However, we found a positive correlation between total preoperative HOAs and M6 postoperative HOAs (*r*^2^ = 0.573; *p* < 0.001).

### 3.4. Efficacy, Stability, and Satisfaction

**Figure 10:**
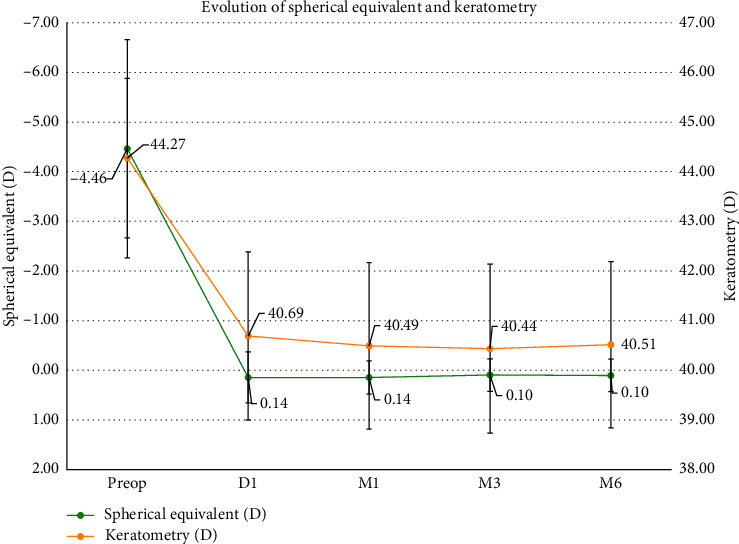
Stability of keratometry and spherical equivalent refraction after LASIK between 1 day and 6 months.

One day after LASIK surgery, the mean refractive spherical equivalent and keratometry were +0.14 ± 0.52 D and 40.49 ± 1.70 D, respectively, and remained stable up to 6 months follow-up (Figure 10).

**Figure 11:**
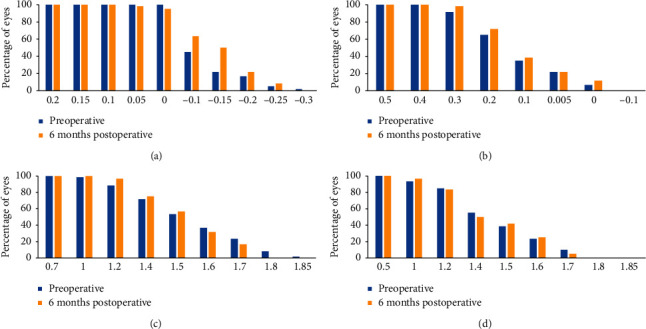
Changes in 90% contrast (a) and 10% (b) uncorrected distance visual acuity and uncorrected contrast sensitivity (c) and uncorrected contrast sensitivity with glare (d) at 6 months of follow-up after LASIK.

Six months postoperatively, 62% of eyes achieved high-contrast UDVA of −0.1 log MAR or better versus 42% CDVA before undergoing LASIK. Uncorrected CCS appeared unchanged 6 months postoperatively compared to corrected CCS in both normal and glare illumination conditions (Figure 11).

**Figure 12:**
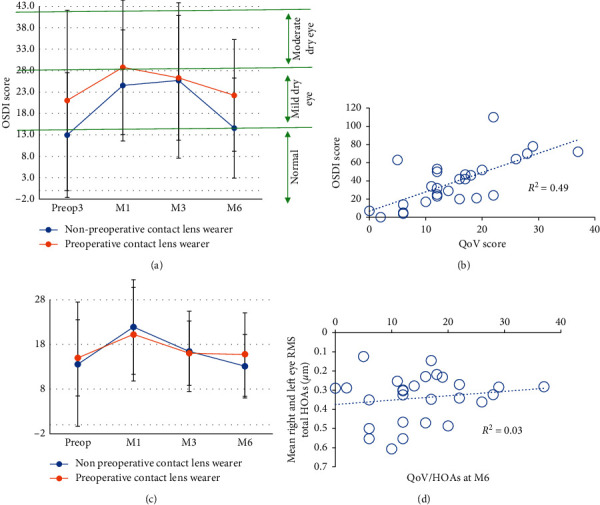
Evolution of QoV (a) and OSDI (c) scores and relationship between the QoV score and the OSDI score (b) and total HOAs (d).

In both populations (preoperative contact lens wearers and nonwearers), QoV score was unchanged from preoperative levels (paired *t*-test, *p*=0.262). The same observation was made for the OSDI questionnaire although it increased and then decreased significantly between preoperative and 6 months postoperative follow-up (Figures 12(a) and 12(c)). Figures 12(b) and 12(d) show that 6 months after LASIK, dry eye symptoms were more related to the QoV score than corneal HOAs, which may explain the lower quality of vision. We found no correlation between the QoV score at 6-month follow-up and the preoperative spherical equivalent (*r*^2^ = 0.0004; *p* < 0.001).

## 4. Discussion

This study aims to explore the midterm post-myopic LASIK refractive surgery clinical results with the WaveLight® Refractive Suite (Alcon® Laboratories Inc., USA) by evaluating changes in ocular anatomical parameters, visual performance, and quality of vision. To the best of the authors' knowledge, there are few comprehensive studies that evaluate anatomical changes of the eye and report outcomes following myopic femtosecond LASIK performed with this Refractive Suite [16, 51–54].

### 4.1. Anatomical Changes

The pachymetry decreased noticeably on D1 and then increased again until 6 months postoperatively. These results could be explained by the fact that Orbscan IIz® (Bausch & Lomb®, USA), which uses a scanning-slit topography system-based measurement of the corneal tomography, largely underestimates the real thickness of the cornea at D1 because of the oedema generated by LASIK [51]. The oedema disappears few days after the LASIK procedure, and the estimation of the pachymetry becomes closer to the real one. Indeed, it is well known that the post-LASIK accuracy of Orbscan II is poorer compared to other devices, but it still gives a good indication on the “pachymetric dynamics.” As a matter of fact, this constitutes a limit of our study. However, Hassan Hashemi and Shiva Mehravaran, in a paper published in the JCRS in 2007, concluded that although Pentacam seems to show better agreement than Orbscan II, especially after refractive surgery, it is not advisable to use different devices interchangeably in every clinical situation [55].

Otherwise, Smadja et al. [51] reported in 2012 that posterior steepening and a shift toward prolateness of the corneal posterior surface were observed very early after myopic LASIK, with a tendency to return toward the preoperative level between 1 month and 3 months. Finally, it has also been described in the literature that there is an epithelial hyperplasia that occurs gradually a few weeks after LASIK [52, 56]. In other words, the increase of pachymetry from D1 to M6 could be partly due to the ability of the epithelium to reshape the operated corneal surface and keep the refraction stable, but without an actual impact on the keratometry if the hyperplasia occurs uniformly in the operated zone, which is the case in the present study.

Figure 4 shows that, in our study, the excimer laser ablated more in the central cornea than the Munnerlyn formula planes by a factor of 16%. Indeed, as the Munnerlyn formula does not consider possible variations in corneal asphericity, actual aspheric treatments induce a slightly different central ablation depth (and ablate more in the peripheries) which allows to maintain a level of postoperative spherical aberration close to preoperative levels [57–60].

One day after LASIK, the corneal asphericity expressed by the *Q* factor became significantly more oblate, and Figure 9 shows that even as the asphericity changed, the spherical aberration calculated on a 5.5 mm pupil increased very slightly (+0.034 ± 0.063). This result is consistent with the increased ablation in the central and peripheral cornea to maintain a low level of positive spherical aberration induced by the surgery [61].

### 4.2. Safety and Predictability

#### 4.2.1. Quality of Vision Outcomes

Figure 5 shows results are consistent with the literature [15, 53]. However, regarding contrast sensitivity, it would have been preferable to study additional spatial frequencies. Indeed, Tuan [15] reported differences in outcomes between different spatial frequencies. We chose to test only the spatial frequency at 12 cpd to ensure the patient remained in comfortable conditions and without overexertion (and thus distortion of the results) due to the long examination sessions (2 hours).

Although there was no difference in the quality of vision outcomes (CDVA and CSB) preoperatively between high (cylinder ≥ 1.5 D) and low astigmatic eyes (cylinder< 1.50 D) except for the CCS (where the high astigmatic eyes CCS was smaller than the low astigmatic one (ANOVA, *p*=0.016)) and no difference in 6 months postoperative residual SE, the high astigmatic eyes had a poorer postoperative CDVA and CCS. This may be due to underoptimized astigmatism ablation profiles and/or nomogram of the excimer. However, we can highlight that the low number of high astigmatic eyes (cyl >1.5 D, only 11 eyes) constitutes a limitation in our study. Indeed, even if the result was statistically significant, we were not sure that we could make robust generalized conclusions for these eyes with astig >1.5 D.

#### 4.2.2. Refractive Spherical Equivalent Outcomes and Astigmatism

The outcomes showed an extremely high predictability and accuracy (Figures 6(a) and 6(b)). These results are consistent with those reported previously by Kanellopoulos and Asimellis [53]. Furthermore, we found that postoperative residual SE was independent from the preoperative degree of myopia and that the high and low astigmatic eyes had the same residual SE. The 6-month postoperative refractive and corneal residual cylinders were low, and we did not find any correlation between the preoperative refractive cylinder and 6-month postoperative cylinder. This suggests that this LASIK technique is predictable in all cases in our sample of eyes. However, it must be noted that our sample did not include eyes with very high amount of astigmatism (maximum included: −3.25 D).

#### 4.2.3. Corneal and Total Aberrations' Analysis on a 5.5 mm Pupil

We note that the evolution profile of coma corresponds to the evolution profile of total, corneal, and internal high-order aberrations (Figure 8 and Table 3). Furthermore, the most important increase in corneal and total HOAs seems to be attributed to the increase of corneal coma (Figure 9). Our results were comparable with those reported by Glydenkerne et al. [62] on a 5 mm pupil. Coma corresponds to a treatment decentration. It is possible then that the increased amount of coma may have been induced by the choice of the centration strategy (i.e., 50% decentration towards the corneal vertex) we planned for all patients.

On a 5.5 mm pupil diameter, the total spherical aberration was well controlled (increased very slightly but significantly (0.034 ± 0.063, *p* < 0.001)) due to the aspheric ablation profiles. These findings indicate that the WaveLight® Refractive Suite (Alcon® Laboratories Inc., USA) aspheric ablation profile seems to limit the increase in postoperative HOAs [61].

Again, our results were comparable with those described by Krueger and Chan [57], but highly different from those reported by Glydenkerne et al. [62] and Bühren et al. [54], where the increase measured on a smaller pupil (5 mm) was, respectively, 0.15 ± 0.084 and 0.153 measured on 6 mm pupil PMMA lenses that received excimer aspheric ablation profile. This is probably due to the less aspheric ablation profile of the excimer laser used.

Moreover, we found that the spherical aberration at M6 was independent from the amount of the corrected myopia (no correlations between total, corneal, and internal spherical aberrations after LASIK and preoperative spherical equivalent (*r*^2^ = 0.03, *p* < 0.001, *r*^2^ = 0.012, *p* < 0.001, and *r*^2^ = 0.009, *p* < 0.001, respectively)), which is consistent with the optimized aspheric profile we mentioned previously.

However, we found a positive correlation between total preoperative HOAs and M6 postoperative HOAs (*r*^2^ = 0.573, *p* < 0.001). Again, this is in favour of a minimal impact of excimer ablation on the increase of HOAs.

Finally, the internal aberrations can be computed by subtracting corneal from total aberration coefficients. Figure 8 and Table 3 show the internal aberrations before and after LASIK surgery. We found a very slight but significant increase in internal ocular aberrations studied (except for internal coma) after LASIK surgery. This increase was higher at D1 postoperatively and then decreased between 1 and 6 months after surgery. Although the LASIK procedure is performed on the anterior surface of the cornea, internal aberrations follow the same evolution profile as corneal and total aberrations. In a previous study [29], Marcos et al. came to the same conclusion. They described their experience in control subjects who had undergone a surgical procedure performed in two different experimental sessions (separated by at least 1 month, as in the surgical eyes) which did not reveal statistically significant changes in the internal aberrations across sessions. This indicated that possible changes across sessions in the accommodative state or decentrations of corneal topography data cannot account for the observed differences in the internal optics found between pre- and post-LASIK results. Therefore, we can conclude that these changes must be attributable to the surgery and specifically to a biomechanical reaction of the posterior surface of the cornea to the surgery [29, 51].

### 4.3. Efficacy, Stability, and Satisfaction

6 months after surgery, 62% of eyes achieved high-contrast UDVA of −0.1 log MAR or better versus 42% CDVA before undergoing LASIK. Uncorrected CCS appeared unchanged 6 months postoperatively compared to the corrected CCS in both normal and glare illumination conditions (Figure 11). LASIK surgery showed good outcomes in terms of efficacy and stability.

Furthermore, in both preoperative contact lens wearers and nonwearers, the QoV score did not change from the preoperative level (paired *t*-test; *p*=0.262). Ocular dryness increased significantly after surgery and returned to the baseline level at M6 (Figures 12(a) and 12(c)). Figures 12(b) and 12(d) show that 6 months after LASIK, QoV score was more related to dry eye symptoms than to corneal HOAs. The quality of vision at M6 did not depend on the preoperative degree of myopia (*r*^2^ = 0.0004; *p* < 0.001) neither on the M6 SE (*r*^2^ = 0.0013; *p* < 0.001). Therefore, we can assume that the patient's quality of vision depends more on the postoperative dry eye disease generated by the laser than on the induced HOAs (which are low in this study) or the patient's initial spherical equivalent correction.

Therefore, we can conclude that LASIK surgery for correction of myopia performed with the WaveLight® Refractive Suite (Alcon® Laboratories Inc., USA) showed good postoperative outcomes and good safety, predictability, efficiency, and stability of postoperative outcomes. These results are likely due to good control of spherical aberration with high performance of aspheric ablation profiles, as well as the use of large optical zones. In addition, our results suggest a change in the shape of the posterior corneal surface as a result of the surgery. Finally, we believe that improvement is required in two main areas: (1) an increase in the predictability of outcomes in eyes with high astigmatism and (2) improved solutions for the issue of ocular dryness.

Our data analysis was limited to 6-month follow-up. Further studies are necessary to investigate potential changes occurring after this time period.

## Figures and Tables

**Table 1:** Demographic data.

	Total	Low cylinder (<1.50 D)	High cylinder (≥1.50 D)
Number of patients	30	24	6
Number of eyes	60	49	11
Right/left	30/30	24/25	6/5
Age (years)			
Mean ± standard deviation	30.4 ± 4.2	30.8 ± 4.2	28.7 ± 4.4
Minimum/maximum	21.3/38.7	21.3/38.7	21.3/36.6
% female/% male	63%/37%	67%/33%	45%/55%
% of the contact lens carrier	70%	78%	36%
Refractive spherical equivalent (D)			
Mean ± standard deviation	−4 5 ± 2.2	−4.6 ± 2.2	−3.8 ± 1.9
Minimum/maximum	−9 3/−0 8	−9.3/−0.8	−6.6/−1.3
Refractive cylinder (D)			
Mean ± standard deviation	−0 8 ± 0.8	−0.5 ± 0.3	−2.1 ± 0.7
Minimum/maximum	0.0/−3.3	0.0/−1.3	−1.5/−3.3

**Table 2:** Differences between high (cylinder ≥ 1.5 D) and low astigmatic eyes (cylinder < 1.50 D) in 90% and 10% corrected distance visual acuity (CDVA), in corrected contrast sensitivity and corrected contrast sensitivity with glare (CCS at 12 cycles per degree), and in corrected sensitivity to blur (CSB).

Safety	1 day postoperative (*n* = 60)			1 month postoperative (*n* = 60)			3 months postoperative (*n* = 60)			6 months postoperative (*n* = 60)		
	Percentage (95% CI)			Percentage (95% CI)			Percentage (95% CI)			Percentage (95% CI)		
	High astigmatism	Low astigmatism	*p* ^*∗*^	Low astigmatism	Low astigmatism	*p* ^*∗*^	High astigmatism	Low astigmatism	*p* ^*∗*^	High astigmatism	Low astigmatism	*p* ^*∗*^
CDVA												
Loss of 3 lines or more (90% contrast)	9%	4%	**0 039** ^*∗*^			**0.037** ^*∗*^			0.137			**0.005** ^*∗*^
Loss of 2 lines (90% contrast)	27%	8%		9%				4%			2%	
Loss of 1 line (90% contrast)	36%	33%		27%	20%		36%	18%		55%	12%	
No loss or gain of lines (90% contrast)	18%	49%		45%	51%		55%	43%		45%	45%	
Gain of 1 line (90% contrast)	9%	6%		18%	27%		9%	29%			33%	
Gain of 2 lines (90% contrast)					2%			6%			8%	
Gain of 3 lines or more (90% contrast)												
Loss of 3 lines or more (10% contrast)	18%	14%	**0.008** ^*∗*^	9%	2%	**0.035** ^*∗*^		2%	0.751^*∗*^		2%	0.08
Loss of 2 lines (10% contrast)	36%	18%		9%				8%			4%	
Loss of 1 line (10% contrast)	36%	22%		18%	22%		18%	16%		36%	18%	
No loss or gain of lines (10% contrast)	9%	37%		36%	41%		36%	27%		18%	27%	
Gain of 1 line (10% contrast)		8%		18%	27%		36%	31%		45%	39%	
Gain of 2 lines (10% contrast)					8%		9%	16%			8%	
Gain of 3 lines or more (10% contrast)				9%							2%	
CCS (12 cpd)												
Loss of 0.4 u.log or more	9%	31%	0.191	9%	6%	**0.001** ^*∗*^	9%	4%	**0.001** ^*∗*^	9%	2%	**0.001** ^*∗*^
Loss of 0.1 to 0.4 u.log	64%	45%		27%	29%		9%	31%		18%	35%	
No loss or gain	9%	6%		27%	18%		18%	16%		36%	18%	
Gain of 0.1 to 0.4 u.log	18%	16%		27%	37%		55%	33%		27%	41%	
Gain of 0.4 u.log or more		2%		9%	10%		9%	16%		9%	4%	
Loss of 0.4 u.log or more (with glare)	—	—		9%	2%	**0.001** ^*∗*^	9%	4%	**0.001** ^*∗*^	9%	6%	**0.001** ^*∗*^
Loss of 0.1 to 0.4 u.log (with glare)	—	—		36%	37%		18%	37%		36%	33%	
No loss or gain (with glare)	—	—		18%	18%		27%	14%		18%	12%	
Gain of 0.1 to 0.4 u.log (with glare)	—	—		18%	37%		27%	37%		27%	43%	
Gain of 0.4 u.log or more (with glare)				18%	6%		18%	8%		9%	6%	
CSB												
Loss of 0.3 D or more					10%	0.197		8%	0.108		8%	0.29
Loss of 0.1 to 0.3 D				9%	29%			39%		9%	45%	
No loss or gain				36%	31%		18%	14%		9%	12%	
Gain of 0.1 to 0.3 D				45%	29%		73%	35%		73%	22%	
Gain of 0.3 D or more				9%	2%		9%	4%		9%	12%	

^*∗*^Significant.

**Table 3:** Evolution of RMS ocular total, corneal, and internal aberrations (*µ*m).

	Preoperative	6 months postoperative	Difference	*p*
Aberrations (RMS in *µ*m)	Mean ± SD	Mean ± SD	Mean ± SD	
Total high-order aberrations (HOAs)	0.237 ± 0.072	0.340 ± 0.135	0.103 ± 0.111	*p* < 0.001^*∗*^
Total coma	0.11 ± 0.062	0.184 ± 0.121	0.074 ± 0.108	*p* < 0.001^*∗*^
Total spherical aberrations (SA4 + SA12)	0.058 ± 0.04	0.093 ± 0.069	0.034 ± 0.063	*p* < 0.001^*∗*^
Corneal high-order aberrations (HOAs)	0.256 ± 0.088	0.355 ± 0.148	0.099 ± 0.115	*p* < 0.001^*∗*^
Corneal coma	0.130 ± 0.081	0.222 ± 0.135	0.093 ± 0.118	*p* < 0.001^*∗*^
Corneal spherical aberrations (SA4 + SA12)	0.155 ± 0.063	0.200 ± 0.109	0.045 ± 0.082	*p* < 0.001^*∗*^
Internal high-order aberrations (HOAs)	0.243 ± 0.064	0.306 ± 0.182	0.063 ± 0.175	*p* < 0.01^*∗*^
Internal coma	0.113 ± 0.053	0.133 ± 0.128	0.02 ± 0.128	*p*=0.26
Internal spherical aberrations (SA4 + SA12)	0.128 ± 0.063	0.159 ± 0.099	0.031 ± 0.10	*p*=0.027^*∗*^

^*∗*^Significant.

## Data Availability

The data set is available at the Rothschild foundation hospital and could be provided if needed.

## Additional Points

*What Was Known.* LASIK surgery for moderate to high myopia, performed with the WaveLight® Refractive Suite, showed good postoperative outcomes, with demonstrated safety, predictability, efficiency, and stability. This is probably due to well-controlled spherical aberration and the use of large optical zones. *What This Paper Adds*. Prior to this paper, there was no exhaustive study performed on the anatomical and visual outcomes after LASIK in moderate to high myopia with the WaveLight® Refractive Suite (Alcon® Laboratories Inc., USA) on a large sample of patients with such an accurate methodology. We have demonstrated that the patient's quality of vision depends more on the postoperative dry eye disease generated by the laser than on the induced HOAs.
